# Development and characterization of a human papillomavirus-based nanoparticle carrier for heterologous vaccine antigens

**DOI:** 10.1038/s41598-025-19009-3

**Published:** 2025-09-25

**Authors:** Yu Wang, Nils Bartelsen, Philipp Arnold, Sandra Mueller-Schmucker, Christoph Weingärtner, Jannis Beutel, Jutta Eichler, Vladimir Temchura, Dominik Damm, Klaus Überla

**Affiliations:** 1 Harald zur Hausen Institute of Virology, University Hospital Erlangen, Friedrich-Alexander-Universität Erlangen-Nürnberg, 91054 Erlangen, Germany; 2 Institute of Functional and Clinical Anatomy, Friedrich-Alexander-Universität Erlangen-Nürnberg, 91054 Erlangen, Germany; 3 Department of Chemistry and Pharmacy, Friedrich-Alexander-Universität Erlangen-Nürnberg, 91054 Erlangen, Germany; 4 Virologisches Institut, Schlossgarten 4, 91054 Erlangen, Germany

**Keywords:** Virus-like particle (VLP), T helper nanoparticles, HIV vacciner, Env trimer, B cell activation, Humoral immune response, HPV-16 L1 capsomers, Copper-free click reaction, Functionalization, DBCO, Biochemistry, Biotechnology, Immunology

## Abstract

The display of a repetitive array of antigens on the surface of nanoparticles has been shown to effectively trigger the activation of cognate B cells and to increase humoral immune responses. Building on extensive knowledge from the development and clinical use of virus-like particle (VLP) vaccines against Human Papilloma Viruses (HPV), we developed an HPV-based nanoparticle carrier presenting Human Immunodeficiency Virus Type 1 (HIV-1) envelope glycoprotein (Env) as a heterologous vaccine antigen on the surface. After purification of the L1 capsid protein of HPV-16 from a bacterial expression system and assembly of L1 proteins into VLPs (L1-VLPs), we conjugated HIV-1 Env trimers with the C-terminus to the surface of the nanoparticles (L1-Env) using copper-free click reactions via Dibenzocyclooctyne (DBCO)-terminated linker molecules. These L1-Env nanoparticles activated Env-specific B cells more efficiently than non-conjugated Env in a B cell receptor-dependent manner. In general, immunization of mice with L1-Env nanoparticles resulted in Env- and L1-specific antibody responses without the need for an adjuvant. L1-Env nanoparticles induced significantly higher Env-specific antibody responses in comparison to a control group that received a mixture of Env trimers and uncoupled L1-VLPs. Concurrently, we observed decreased L1-specific antibody responses in comparison to mice that were immunized with uncoupled L1-VLPs suggesting partial shielding of L1 epitopes by conjugated Env trimers. In summary, HPV-based nanoparticles provide an attractive novel approach for combinatorial vaccines against sexually transmitted pathogens.

**Supplementary Information:**

The online version contains supplementary material available at 10.1038/s41598-025-19009-3.

## Introduction

Vaccination plays a crucial role in prevention of disease and improvement of global health. Vaccine design encompasses several key aspects, including antigen selection, adjuvants, delivery strategies, and scalable production systems^1,2^. While attenuated vaccines may pose safety risks in immunocompromised patients due to potential reversion to pathogenic forms, subunit vaccines, despite their higher safety profile, often compromise on efficacy. Consequently, the development of subunit vaccine formulations that balance safety and effectiveness requires ongoing innovation and exploration. In recent years, advances in nanotechnology have introduced new approaches to nanomedicine and vaccine development^3–5^. Compared to traditional vaccines, nanoparticular vaccines may provide significant advantages for antigen delivery, dosing regimens, administration routes, adjuvant integration, and overall vaccination outcomes. The use of nanoparticles as presentation platforms, displaying highly repetitive antigen arrays on their surfaces, is particularly effective in enhancing the immunogenicity of subunit vaccines.

Most protein antigens are too small to be effectively internalized by antigen-presenting cells (APCs) and are prone to degradation at the injection site, limiting their ability to induce protective immunity^6^. Nanoparticles (NPs), ranging in size from 10 to 1000 nm, resemble pathogenic microorganisms and can trigger endocytosis mechanisms, making them particularly suitable for APC uptake^7^. While phagocytosis, a form of endocytosis that engulfs larger particles, is primarily performed by specialized cells like macrophages, nearly all cells can internalize NPs via endocytosis. The physicochemical properties of NPs - such as size, shape, surface charge, and surface chemistry - play a crucial role in regulating cellular uptake efficiency^8^. Additionally, the size of macromolecules influences their entry into lymphatic vessels, with those between 20 and 200 nm being more likely to traverse the lymphatic interstitium^9^. Thus, an effective strategy to enhance the immunogenicity of protein antigens is the use of NP platforms that present repetitive antigen arrays on their surface. This approach has been shown to improve APC uptake, lymph node (LN) trafficking, and B cell activation by increasing particle size and avidity. The ability of NPs to interact with immune components and to stimulate both humoral and cellular immune responses renders them ideal for vaccine design.

Numerous strategies have been pursued to produce nanoparticle-based platforms conjugated with HIV-1 Env trimers for preclinical trials. On the one hand, Env was genetically fused to protein subunits that could self-assemble into nanoparticulate structures upon release from the producer cells, i.e. ferritin^10^ or two-component capsid cores^11,12^. On the other hand, Env trimers were conjugated to the surface of pre-existing nanoparticles by tag-based or tag-free chemical coupling strategies. Inorganic iron oxide nanoparticles were conjugated with Env trimers via EDC/Sulfo-NHS chemistry targeting primary amines on a C-terminal KG_4_ tag^13^. Env trimers were also coupled to the surface of inorganic calcium phosphate nanoparticles harnessing C-terminal aldehyde tags and copper-dependent click reaction with a bi-specific linker molecule^14^. Moreover, silica nanoparticles were functionalized via maleimide groups and recombinant cysteine moieties on Env^15^. A high proportion of studies described the production, characterization and usage of organic liposomes conjugated with Env trimers^16^. Here, coupling strategies included tag-free EDC/Sulfo-NHS crosslinking^17,18^, non-covalent His-tag/Ni-NTA interaction^19^ or maleimide linkage^20^. Furthermore, Env trimers were coupled to the surface of microspheres via coiled-coil interactions^21^ and displayed on DNA origami nanoparticles via hybridization of outward-facing ssDNA overhangs to a complementary peptide nucleic acid^22^. Even though most of these nanoparticle platforms demonstrated promising results in small animal models, the translation into clinical studies is challenging, since each organic or inorganic nanoparticle component as well as the coupling reagents need to be produced under good manufacturing practice (GMP) conditions and overcome extensive preclinical and clinical safety tests.

The human papillomavirus (HPV) L1 protein, when assembled into virus-like particles (L1-VLPs), triggers the production of neutralizing antibodies and serves as the basis of all commercially available HPV vaccines. Continuous global surveillance of HPV vaccination programs consistently validates both the efficacy and safety of these vaccines. This renders nanoparticle delivery systems based on HPV L1-VLPs highly acceptable for widespread use^23^. Additionally, the vaccine Cecolin^®^, produced through an *E. coli* expression system, received World Health Organization (WHO) prequalification in recent years. This method of production is more cost-effective and scalable, with endotoxins effectively removed through chromatography, ensuring the final product meets biosafety standards^24^. Leveraging the self-assembling properties of HPV L1-VLPs for HIV vaccine design is therefore a logical and innovative strategy.

In this study, we designed a novel nanoparticle carrier platform which is based on proteinaceous HPV VLPs. To this end, we conjugated HPV-16 L1-VLPs, which are composed of pentamers of the structural protein L1, with HIV-1 Env trimers via copper-independent click reactions. Beside the potent activation of Env-specific B cells, the observed humoral immune responses in mice suggest that these L1-Env nanoparticles provide an attractive novel approach for combinatorial vaccines tackling two major sexually transmitted diseases.

## Methods

### Recombinant HPV-16 L1 protein expression in *Escherichia coli (E.coli)*

The nucleotide sequence coding for a full-length HPV-16 L1 protein (GenBank accession number KC166220.1) was used as reference. A sequence codon-optimized for *E.coli* and lacking the nucleotides for the first twenty-nine N-terminal amino acids after the start codon (Figure S1) was synthesized and inserted into a modified pET-100 D-TOPO™ vector (Thermo Fisher, Waltham, MA, USA), from which the 6xHis-tag, T7-tag (gene 10 leader) and Xpress™ tag/enterokinase fragment were removed. BL21 (DE3) *E. coli* cells (Thermo Fisher, Waltham, MA, USA) were transformed with this construct. The bacteria were grown at 37 °C and 200 rpm until they reached an optical density at 600 nm (OD600) of 0.5. At this point, 0.5 mM isopropyl-β-d-thiogalactopyranoside (IPTG; Thermo Fisher, Waltham, MA, USA) was added to induce protein expression and the bacteria were further incubated at 25 °C for 20 h. The bacteria were then lysed by sonication (Branson Ultrasonics™ S-450 A, USA). The cell debris was pelleted (8000 rpm, 30 min, 4 °C) and the supernatant containing soluble L1 proteins was collected for further purification.

### Purification and assembly of HPV-16 L1-VLPs

In a first step, L1 proteins were precipitated from bacterial supernatant by dropwise addition of saturated ammonium sulfate solution (AS, Carl Roth, Karlsruhe, Germany) up to a final AS concentration of 40% (v/v). The resulting L1 pellet was resuspended in 20 mM Dithiothreitol (DTT)-containing phosphate buffer (PB; Carl Roth, Karlsruhe, Germany) at pH 7.5. Subsequently, L1 protein was further purified through chromatography using SP Sepharose (Cytiva, Marlborough, MA, USA) with gradient elution (from 0 M to 0.5 M NaCl) to remove impurities and the eluted fractions were collected in PB containing 0.75 M NaCl. The self-assembly of L1-VLPs was facilitated by the removal of the reductant and the adjustment of the pH from 7.5 to 6.0 via dialysis (SnakeSkin™ Dialysis Tubing, 10 K MWCO, Thermo Fisher, Waltham, MA, USA). Finally, VLPs were purified via size-exclusion chromatography (SEC) utilizing a Sephacryl^®^ S-300 column (Cytiva, Marlborough, MA, USA). The whole disassembly-reassembly process was monitored via negative-stain transmission electron microscopy (TEM) as previously described^25^ with the following exceptions: L1-VLPs, aggregates or pentamers were pipetted onto a freshly negative glow discharged continuous carbon grid (science Service Munich, Munich, Germany) and then washed with 2% uranyl acetate solution twice. Subsequently, the grids were inserted into a JEOL 1400Plus TEM (JEOL, Munich, Germany) operating at 120 kV for image acquisition.

### Functionalization of L1-VLPs

The L1-VLP preparation was treated with a tenfold molar excess of TFP Ester-PEG4-Dibenzocyclooctyne (DBCO) (EZ-Link™, Thermo Fisher, Waltham, MA, USA) to functionalize the VLP surface with DBCO groups (L1-DBCO). After 2 h incubation at room temperature (RT), non-reacted linker molecules were removed by ultrafiltration (Amicon^®^ Ultra-15, 100 kDa MWCO, Merck, Germany). To characterize the functionality of the click-on reaction targeting the terminal DBCO groups, an unrelated peptide (Fluo-EIAALEREIAALEREIAALER-Aoa-Aoa-Lys-(N_3_)-NH_2_, synthesized by Dr. Jannis Beutel) containing an azide ester on the C-terminus and Fluorescein Isothiocyanate (FITC) on the N-terminus was added to the L1-DBCO sample at 20-fold molar concentration.

### Orthogonal, covalent coupling of HIV-1 Env trimers with L1-DBCO

HIV-1 Env trimers with a C-terminal aldehyde-tag (LCTPSR; Env-Ald_6_) for orthogonal conjugation were produced as described elsewhere^14^. Briefly, Env proteins were expressed in 293F suspension cells (Thermo Fisher, Waltham, MA, USA) by transient transfection with the plasmid pBG505-NFL2P-gp140-Ald_6_^14^. The cells were co-transfected with a plasmid coding for formylglycine-generating enzyme (FGE) to optimize post-translational aldehyde conversion. Env-Ald_6_ trimers were purified from the cell culture supernatant via lectin affinity chromatography (Agarose-bound *Galanthus nivalis* lectin, Vector Laboratories Inc., Burlingame, CA, USA). Subsequently, Env-Ald_6_ was incubated overnight at 37 °C and 400 rpm with aminooxy-PEG3-azide linker (Broadpharm, San Diego, CA, USA) under oxime reaction conditions (250 mM NaAC, pH 4.5) to yield azide-terminated Env-Ald_6_ (Env-N_3_). After a buffer exchange by ultrafiltration (Amicon^®^ Ultra-15, 100 kDa MWCO, Merck, Germany), Env-N_3_ was coupled to L1-DBCO via a non-catalyzed click reaction (4 °C, 18 h). Any uncoupled Env-N_3_ proteins were separated by an additional SEC purification step using a Sephacryl^®^ S-300 column (Cytiva, Marlborough, MA, USA).

### Ethical statement for preclinical trials

This study included female wild-type (WT) mice (C57BL/6NRj, Janvier, Le Genest-Saint-Isle, France) and in-house bred, HIV-1 Env-specific B cell receptor-transgenic mice of both sexes (PGT121 mice^26^ kindly provided by Dr. Michel Nussenzweig, Rockefeller University, New York City, NY, USA). All mice were accommodated in ventilated cages at the animal facility (Franz-Penzoldt-Center) of the Faculty of Medicine, FAU (Erlangen, Germany) and handled as recommended by the Federation of European Laboratory Animal Science Association and as approved by the Government of Lower Franconia (License 55.2.2-2532-2-1199). Mice were euthanized after experiments or for organ donations by CO_2_ treatment with subsequent cervical dislocation. The initial flow rate of CO_2_ was 20% of the cage volume per minute. The mice usually lost consciousness within the first minute. After 10 min treatment, the flow rate was increased up to 70% volume per minute for 3 min. Cervical dislocation was done afterwards. During experimental procedures, the mice were anesthetized with isoflurane in a separate flow chamber using an automated vaporizer (4% air volume, flow rate: 0.9 L/min for induction; 1.4% air volume, flow rate: 0.9 L/min for maintenance).

### B cell activation in vitro

Wild-type (WT) and Env-specific (PGT121) B cells were isolated from spleens of C57BL/6NRj and B cell receptor-transgenic mice respectively, with a MACS-based isolation kit for murine naïve B cells following the manufacturer’s instructions (Miltenyi Biotec, Bergisch-Gladbach, Germany). Afterwards, 2 × 10^5^ cells were seeded into U-bottom 96-well cell culture plates (Greiner, Kremsmünster, Austria) in 100 µL of R10 medium (RPMI 1640, 10% FCS, 1% Penicillin-Streptomycin, 10 mM HEPES, 2 mM L-glutamine, 50 µM 2-mercaptoethanol), respectively. Nanoparticle dilutions in a total of 100 µL R10 medium were added and the plates were incubated for 24 h at 37 °C with 5% CO_2_. Lipopolysaccharide (LPS; Merck, Germany) and HIV-1 VLPs were used as polyclonal and Env-specific positive controls. The cells were then stained with anti-CD19-Qdot655, anti-CD86-FITC and fixable viability dye eFluor450 (all Thermo Fisher Scientific, Waltham, MA, USA). Surface expression levels of activation markers of living B cells were evaluated with a spectral flow cytometer (Cytek Northern Lights, USA) and analyzed with FlowJo software (BD Biosciences, USA).

The method for producing lentiviral HIV-1 VLPs has been described previously^27^. Briefly, 293T cells were transfected with equal amounts of the plasmids pHgpSyn and pConBgp140GC/D, coding for HIV-1 Gag/Pol and a membrane-anchored Env glycoprotein respectively, using 1.5 µg of polyethylenimine per 1 µg of DNA. After 2–3 days, VLPs were purified from the supernatant of the transfected cells by ultracentrifugation (35% sucrose cushion, 2.5 h, 28,000 rpm, 4 °C).

### Immunization and blood collection

Mice, aged 6 to 8 weeks, were immunized twice in a 4-week interval with either high, medium, or low doses of L1-Env nanoparticles or an uncoupled control (Table 1). The vaccine candidates were injected intramuscularly (i.m.) in both hind legs under isoflurane (CP-Pharma, Burgdorf, Germany) anesthesia in a total volume of 60 µL per mouse. Blood samples were taken from the retrobulbar venous plexus using minicaps^®^ (Hirschmann, Eberstadt, Germany). Blood was collected in BD Microtainer^®^ collection tubes (Becton Dickinson, Franklin Lakes, NJ, USA). After a 5 min centrifugation at 5000 rpm, the sera were isolated and stored at −20 °C. All mice were euthanized after the last blood collection.

**Table 1:** Immunization doses for L1-Env nanoparticles. Mice were immunized with 60 µL of L1-Env nanoparticle preparations containing the indicated concentrations of Env and L1 for the high, medium, and low dose groups. Two independent batches of nanoparticles were produced for the first and second immunizations. Control groups received L1-VLPs and uncoupled Env with matching amounts of each immunogen.

	1. Vaccination		2. Vaccination	
	L1 [µg/mL]	Env [µg/mL]	L1 [µg/mL]	Env [µg/mL]
High dose	900	20	100	2
Medium dose	90	2	10	0.2
Low dose	9	0.2	1	0.02

### Analysis of humoral immune responses

To measure the levels of Env-specific or HPV-specific antibodies in the sera of vaccinated mice, 150 ng of BG505 Env protein or 500 ng of L1-VLPs in 100 µL of coating buffer were added to each well of a white, high-binding, flat-bottomed 96-well assay plate (Greiner, Kremsmünster, Austria). Following overnight incubation at 4 °C, the wells were blocked with 5% skimmed milk in PBS/T (PBS containing 0.05% of Tween-20). Afterwards, 100 µL of Murine sera diluted 1:1250 in 2% skimmed milk were added for detection. The plate was then washed three times. Next, HRP-conjugated anti-mouse IgG (Dianova, Hamburg, Germany), diluted 1:5000 in PBS/T, was added and incubated for 60 min at RT. Finally, the plate was washed again, and 70 µL of a ECL solution (0.1 M Tris, 250 mg/L luminal sodium salt, 22 mg/L p-coumaric acid, 3.4 µL 30% H_2_O_2_ ad 10 mL H_2_O) were added before measuring luminescence using the Victor X4 multi-label plate reader (PerkinElmer, Waltham, MA, USA).

## Results

### Expression, purification and characterization of HPV-16 L1-VLPs

**Fig. 1:**
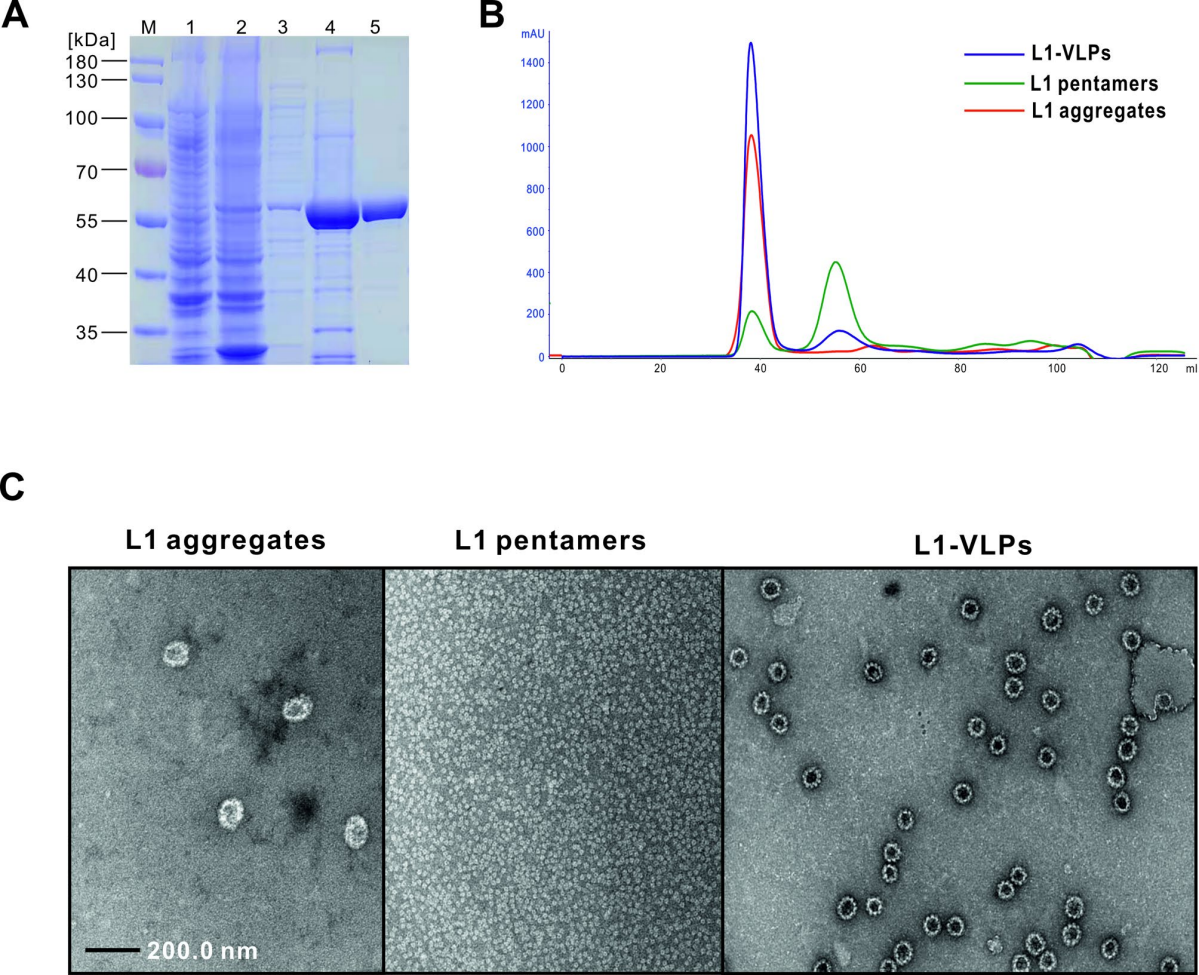
Preparation and characterization of L1-VLPs. (**A**) Analysis of the expression and purification of HPV-16 L1 protein, visualized by SDS-PAGE and Coomassie blue staining. Lane 1 shows the sample without IPTG induction during *E. coli* expression, while Lane 2 shows the sample with IPTG induction. Lanes 3–5 represent the sequential purification steps, including samples obtained after ammonium sulfate precipitation, cation-exchange chromatography and SEC. (**B**) The in vitro disassembly-reassembly process was monitored using SEC. (**C**) TEM images of purified L1 aggregates, L1 pentamers and L1-VLPs.

We deleted the nucleotides encoding the first twenty-nine amino acids after the initial methionine of the HPV-16 L1 open reading frame to enhance the expression of the recombinant protein in *E. coli* (Figure S1)^28^. After bacterial transformation, L1 expression was induced by addition of 0.5 mM IPTG (Fig. 1A). Since L1 is not released into the supernatant through the secretory pathway, bacteria were lysed for subsequent purification of L1. The resulting lysate contained a multitude of host proteins, cell debris, nucleic acids, pigments, and other small molecules. Balancing the purity of the target protein with cost, efficiency, and scalability of the purification process is a common technical challenge in the development of recombinant protein vaccines from *E. coli*. As an initial purification step, a 40% AS concentration was utilized to salt out the target protein. After precipitation, the AS solution was separated and the precipitated protein was re-dissolved in phosphate buffer. SDS-PAGE analysis showed that most of the host proteins were effectively removed (Fig. 1A). Additionally, the protein solution became visibly clearer (data not shown), indicating that bacterial pigments and nucleic acids were also successfully eliminated. Soluble L1 proteins were adsorbed by a cation exchange column at pH 7.5 and subsequently purified and concentrated through salt gradient elution. Concurrently, endotoxins from bacteria, which bear strong anionic charges, passed through the column and were separated. The endotoxin levels in protein samples from different production batches, measured using the Chromogenic LAL Endotoxin Assay Kit (GenScript, Piscataway, NJ, USA), were all below 0.1 EU/mL (data not shown). L1 purity was assessed by Coomassie blue staining with the most prominent bands observed at 55 kDa (Fig. 1A). Importantly, all procedures are scalable and well-established, ensuring reliable and reproducible outcomes.

Recombinant L1 proteins do not spontaneously self-assemble into VLPs during intracellular expression in bacteria. Instead, a disassembly-reassembly process in vitro is typically required. Irregular L1 aggregates that accumulate in *E. coli* were dissolved into soluble pentamers under the influence of the reducing agent DTT. The reducing agent was gradually removed through dialysis and the buffer environment was transitioned to one that is weakly acidic as well as high in salt concentration to promote VLP reassembly. The alteration in particle size was evident by SEC (Fig. 1B). The shifting of elution peaks of the particles in the presence or absence of DTT exchange reflected the disassembly-reassembly process in vitro. Electron microscopy (EM) observations provided definitive morphological evidence of the nanoparticles (Fig. 1C). These observations revealed distinct morphological differences including protein aggregates directly produced by bacteria, pentamers spanning approximately 10 nm in diameter formed after disassembly, and the eventual self-assembled, uniform, regular, and hollow VLPs (~ 50 nm in diameter).

### Conjugation of Env trimers with L1-VLPs

**Fig. 2:**
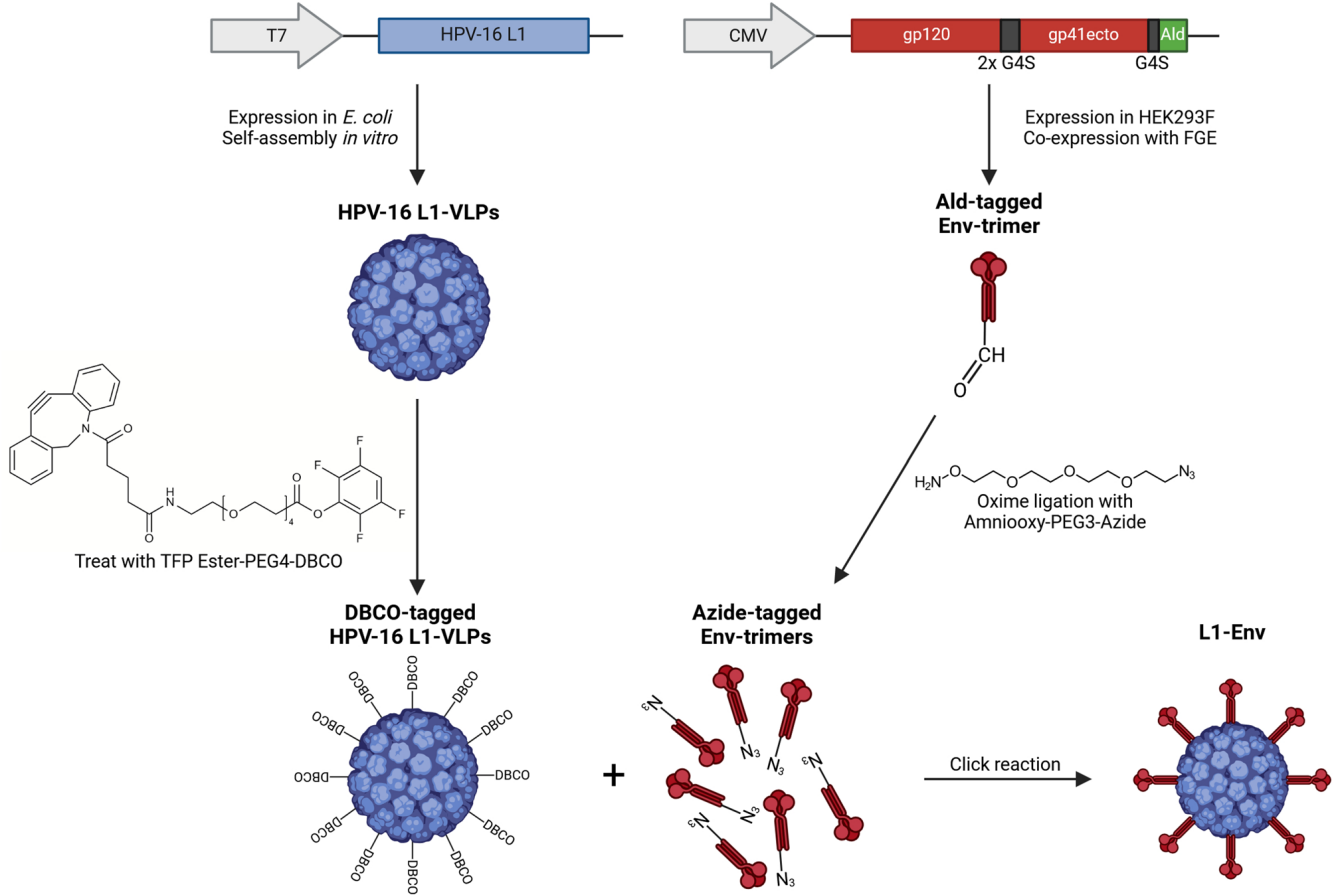
Technical route for constructing L1-Env nanoparticles. ﻿﻿﻿HPV-﻿16 L1 proteins were expressed in E. coli and subsequently assembled into L1-VLPs in vitro. The L1-VLPs were incubated with TFP-Ester-PEG4-DBCO to attach DBCO to the particle surface, allowing conjugation via copper-free click chemistry. Aldehyde-labeled Env trimers were expressed in HEK293F cells while co-expressing FGE to enhance the conversion of cysteine to formylglycine. Subsequently, the trimers were ligated with aminooxy-PEG3-azide to label them with reactive azide groups at the C-terminus. Finally, the DBCO-labeled VLPs and the azide-labeled Env trimers were conjugated via click chemistry.

In order to produce L1-Env nanoparticles, a potential strategy might involve the chemical functionalization of the L1-VLP surface with a click-on ligand. Subsequently, the antigen of interest that bears another complementary ligand can be densely conjugated onto the VLP surface through this reaction (Fig. 2). In a previous study we have developed an orthogonal coupling method that allows Env trimers to be displayed on the surface of nanoparticles in a configuration similar to the native viral arrangement. Env trimers with a C-terminal aldehyde-tag were produced in HEK293F cells with co-expression of FGE to enhance the conversion of cysteine to formylglycine. Subsequently, the trimers were ligated with aminooxy-PEG3-azide, labeling them with reactive azide groups at the C-terminus. We have demonstrated that Env trimers coupled in this manner are more effective at activating Env-specific B cells (in vitro) and inducing Env-specific antibody responses (in vivo) compared to random linkage^14^.

Treatment of L1-VLPs with a TFP-PEG4-DBCO linker leads to TFP esters that exhibit specificity towards primary amines and form stable amide bonds (L1-DBCO). The efficiency of the conjugation reaction could be assessed by quantifying the absorbance of the protein at 280 nm and the absorbance of the DBCO group at its excitation maximum (309 nm). The degree of labeling, estimated to be approximately 5 DBCO esters per monomeric L1 protein, was determined using the formula: $\:\frac{{DBCO}}{{L1}} = \frac{{A309 \times \:M_{{DBCO}} }}{{(A280 - 1.089 \times \:A309) \times \:M_{{L1}} }}$.

**Fig. 3:**
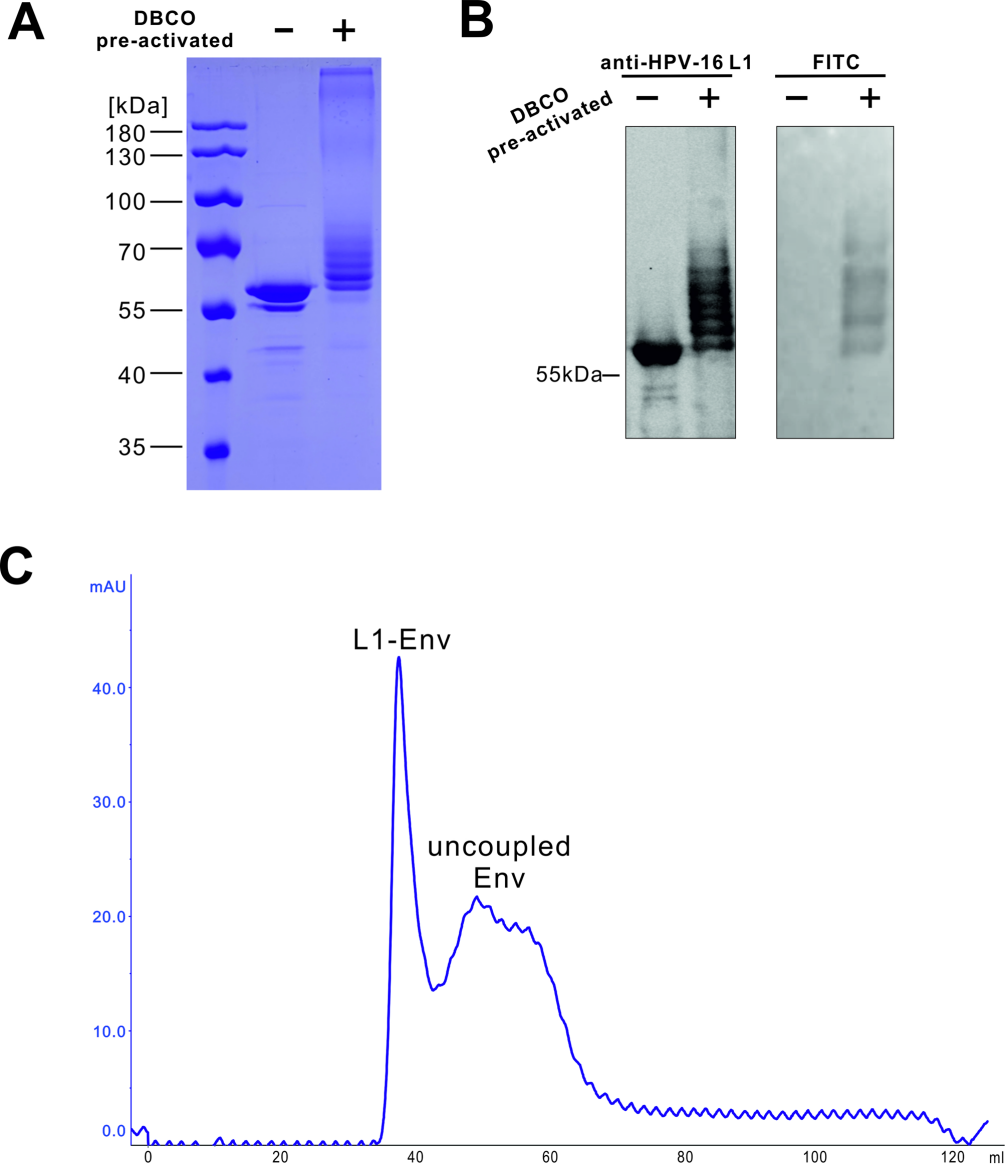
Covalent coupling of L1-VLPs. DBCO pre-activated L1-VLPs captured azide-labeled unrelated peptides. Coupling efficiency was analyzed via gel electrophoresis followed by Coomassie staining (**A**). (**B**) The antigenicity of L1-DBCO (+) compared to L1 alone (-) was assessed by western blotting (left panel) using an HPV-16 L1 antibody (CAMVIR-1, Santa Cruz Biotechnology, USA). Likewise, L1 was pre-activated with DBCO groups and conjugated to a fluorescent azide-linker to prove the conjugation efficiency. Fluorescently conjugated L1 (+) and a control without DBCO pre-activation (-) were analyzed after SDS-PAGE by fluorescence imaging (right panel). Complete gel images are shown in Supplementary Figure S2. (**C**) After N_3_-modified Env trimers were covalently linked to the surface of the L1-VLPs, unbound Env was efficiently separated by SEC.

A specifically modified peptide (Fluo-EIAALEREIAALEREIAALER-Aoa-Aoa-Lys-(N_3_)-NH_2_) with a molecular weight of approximately 3 kDa and a FITC-tag was utilized to evaluate the capability of L1-DBCO to capture the N_3_ group. Upon covalent linkage, changes in the molecular weight of the L1 protein were observed via SDS-PAGE following interaction with the peptide (Fig. 3A). The conjugated L1 appeared as a ladder of bands, with apparent molecular weights exceeding the 55 kDa band of unconjugated L1 capsid protein. Each band above the unconjugated band was indicative of one molecule of peptide attached per L1 monomer, with subsequent bands representing further peptide attachments per L1 molecule. Additionally, the antigenicity of the shifted bands was assessed through Western blotting. All weight-increased components reacted specifically with the anti-HPV-16 L1 antibody and exhibited green fluorescence when visualized under a fluorescence camera, emanating from the FITC-tag of the peptide (Fig. 3B). Multiple bands with different sizes confirmed the potential of the platform to successfully couple azide-labeled antigens with high density. Finally, linker-bound Env (Env-N_3_) was likewise coupled to the surface of L1-DBCO. After the incubation time of the Click-on reaction, unbound Env trimers were effectively separated using SEC to eliminate any potential interference from free Env in subsequent immunoassays (Fig. 3C).

### Activation of Env-specific B cells by L1-Env nanoparticles

**Fig. 4:**
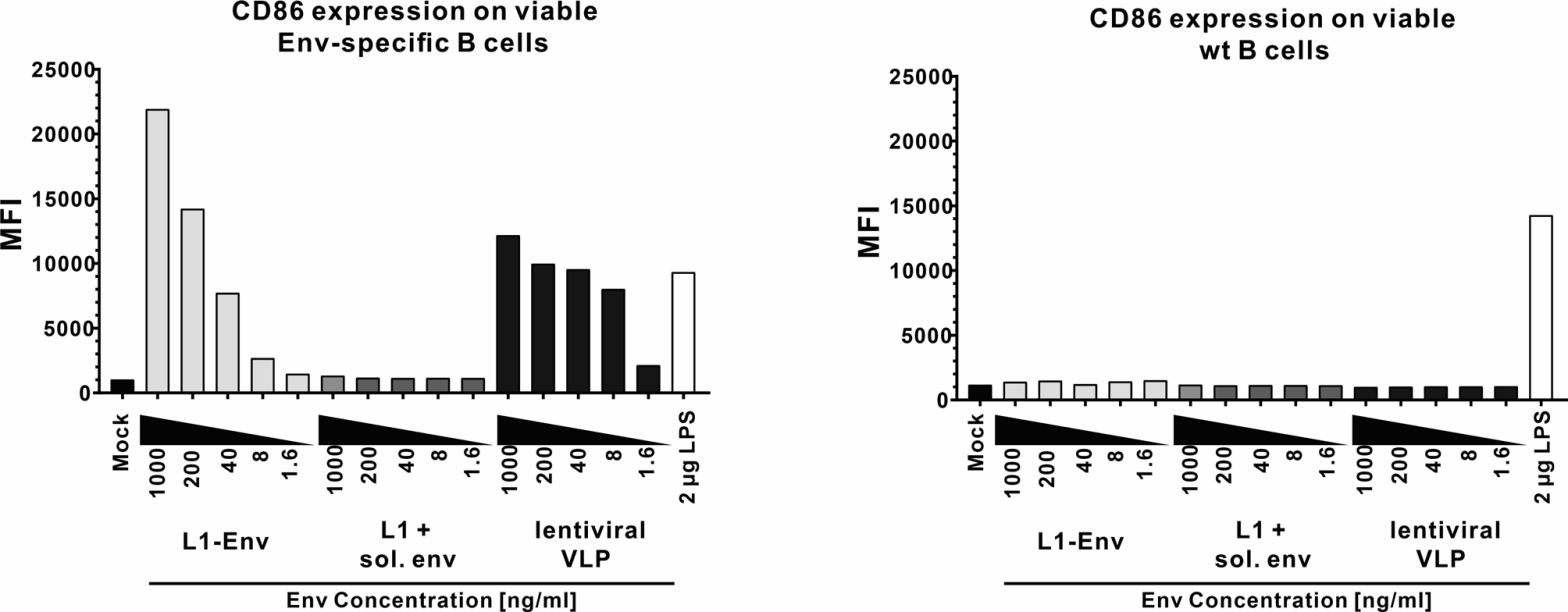
CD86 expression after in vitro stimulation of naive B cells by L1-Env nanoparticles. Naive Env-specific PGT121 (left panel) or wild-type (wt, right panel) B cells (2 × 10⁵) were incubated for 18 h with L1-Env and lentiviral VLPs at various dilutions normalized to their Env concentration (1.6–1000 ng/ml). Lentiviral VLPs were used as a positive control for BCR-dependent stimulation of Env-specific B cells, and 2 µg/ml LPS served as a positive control for polyclonal stimulation of B cells. Negative controls included unstimulated (mock) cells or cells incubated with a mixture of L1-VLPs and soluble Env at concentrations ranging from 1.6 to 1000 ng/ml of each protein. After 18 h, cells were stained for viability, and B cell activation was assessed by measuring the upregulation of CD86 on CD19 + cells via flow cytometry.

A repetitive display of Env on the surface of L1 nanoparticles should lead to enhanced activation of Env-specific B cells due to more efficient crosslinking of B cell receptors (BCR). In order to test this hypothesis, both Env-specific and wild-type B cells were isolated from BCR-transgenic PGT121 mice and C57bl/6NRj mice, respectively. Both types of B cells were then incubated with different dilutions of L1-Env nanoparticles (1.6–1000 ng/mL Env concentration). As a negative control for BCR crosslinking, L1-VLPs mixed with non-coupled Env trimers were used. Lentiviral VLPs presenting HIV Env were used as an antigen-specific positive control^27^. A polyclonal B cell activation control was incubated with LPS. After 18 h of incubation, the B cells were analyzed for viability and upregulation of the B cell activation marker CD86 via flow cytometry (Fig. 4). CD86, expressed by resting naive B cells at low levels, is upregulated upon cognate antigenic binding to the BCRs or polyclonal stimulation via LPS receptor^29^. L1-Env nanoparticles induced a dose-dependent upregulation of the activation marker CD86 in Env-specific B cells, peaking at 1000 ng/mL Env. At the same time, incubation of Env-specific B cells with L1-VLPs combined with uncoupled Env protein did not induce CD86 upregulation, clearly indicating that coupling of Env to the surface of L1-VLPs resulted in efficient BCR crosslinking (Fig. 4). Importantly, incubation of wild-type B cells with L1-Env nanoparticles did not result in CD86 upregulation. This observation indicated a BCR-specific activation and also revealed that both L1-VLP and L1-Env nanoparticle preparations were free from polyclonal B cell activators. All applied dilutions of nanoparticles resulted in similar viability of B cells in vitro (data not shown).

### Characterization of the antibody response to L1-Env nanoparticles

Lastly, we investigated whether L1-Env nanoparticles could induce L1- and Env-specific humoral immune responses in a dose-dependent manner. To this end, mice were vaccinated at weeks 0 and 4 with high, medium or low doses of L1-Env nanoparticles (Table 1), or with uncoupled controls containing L1-VLPs and soluble Env in matching concentrations. Blood samples were collected in week 3 and 6, and the serum was analyzed for L1- and Env-specific IgG antibodies.

**Fig. 5:**
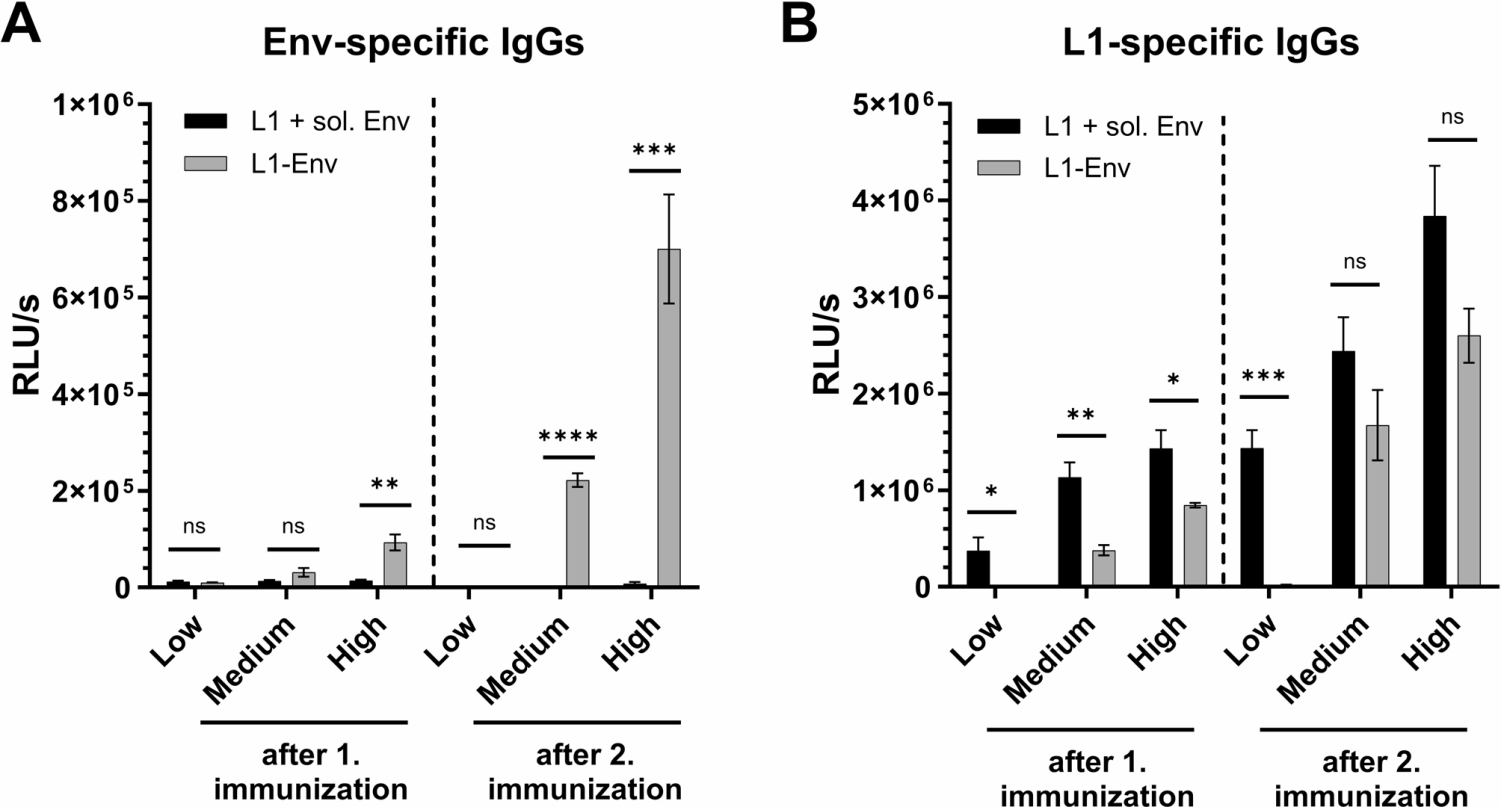
Env- and L1-specific IgG serum concentrations after immunization with L1-Env. Mice were vaccinated at week 0 and 4 with 60 µL of either low, medium, or high doses of L1-Env nanoparticles or corresponding low, medium, or high doses of L1-VLPs mixed with soluble Env as controls (see Table 1 for antigen amounts). Blood samples were drawn at week 3 and 6. (**A**) The levels of Env-specific IgG antibodies in the sera were evaluated using a serum ELISA, with results expressed as relative light units per second (RLU/s). Data are presented as the mean ± standard error of the mean (SEM) for *n* = 4. Statistics were performed using Mann-Whitney tests. (**B**) The levels of L1-specific IgG antibodies in the sera (expressed as RLU/s) are shown, with each column representing the mean ± SEM of the respective group. Statistical differences between groups were assessed using the Mann-Whitney test. All significance levels are indicated as follows: * *p* < 0.05; ** *p* < 0.01; *** *p* < 0.001; **** *p* < 0.0001.

Intramuscular immunization with L1-Env nanoparticles triggered a dose-dependent Env-specific IgG response after the first immunization, with an enhanced response following the second immunization. However, an Env-specific IgG response was only observed in the medium- and high-dose groups. The uncoupled control failed to induce Env-specific IgG responses across all doses, even after the second immunization, highlighting the strong increase in immunogenicity provided by the conjugation of Env to L1-VLPs (Fig. 5A).

In the medium- and high-dose groups, both L1-Env nanoparticles and the uncoupled controls induced a dose-dependent L1-specific IgG response after the first immunization, which was increased after the boost (Fig. 5B). Interestingly, despite using equal concentrations of L1 for the high, medium, and low doses, the L1-specific IgG response was consistently lower in mice immunized with L1-Env compared to those receiving the uncoupled L1-VLPs. This suggests a potential shielding effect caused by the functionalization of the L1-VLPs with Env trimers. The most pronounced shielding effect was seen in the low-dose group, where no L1-specific IgGs were detected after both immunizations, while the uncoupled control group exhibited a detectable L1-specific response that was further amplified after the second vaccination.

## Discussion

HIV-1 has spread across the globe for more than forty years, but a vaccine that is able to elicit protective immunity is still missing^30^. It is widely believed that the elicitation of broadly neutralizing antibodies (bNAbs) by a vaccine could mediate protection against HIV-1 infection^31,32^. So far, numerous clinical vaccine trials using monomeric forms of the HIV-1 surface glycoprotein (Env) failed to consistently elicit bNAbs. During the last decade, soluble, recombinant forms of the Env trimer stabilized in the pre-fusion conformation and bearing multiple epitopes for bNAbs were rationally designed^33–36^. More recently, germline-targeting antigens and sequential immunization strategies were developed to overcome the poor recognition of the Env trimers by B cells expressing germline-encoded BCRs^37,38^.While some of these immunogens are currently tested in phase-1 clinical studies (NCT03816137; NCT04224701; NCT04177355)^39^, limitations in previous preclinical trials were marked by poor immunogenicity and the induction of autologous, but not heterologous or even broadly-neutralizing, antibody responses^40–43^.

The BG505 NFL trimer used in the current study is one of the first “native-like” immunogens that offered substantial immunological advantages over earlier Env designs^44^. Prior subunit vaccines, such as monomeric gp120, typically induced antibodies against easy-to-neutralize Tier-1 viruses or non-neutralizing responses. In contrast, native-like BG505 trimers may foster autologous Tier-2 neutralizing antibodies in guinea pigs, rabbits and non-human primates, marking significant progress in HIV vaccine research^45^. Moreover, BG505 NFL trimers display multiple conserved bNAb epitopes. All major classes of bNAbs isolated from HIV-infected patients bind effectively to BG505 trimers, confirming the presence and antigenic integrity of these epitopes^36^. However, recombinant Env trimers alone fail to induce robust immune responses. Consistent with prior findings, control groups immunized with soluble trimer proteins in this study produced barely detectable Env-specific antibody levels (Fig. 5A). Independent of further optimization of the conformation of the Env trimer, improving the immunogenicity of the Env immunogens in general by next-generation adjuvants or advanced delivery systems, such as recombinant nanoparticles, may be required for the development of successful HIV vaccines.

As one potential strategy for developing a preventative HIV-1 vaccine, it was shown that nanoparticles site-specifically conjugated with the C-terminus of stabilized Env trimers could improve the immunogenicity in vivo and in vitro and promoted the masking of potential non-neutralizing epitopes, while most of the epitopes for bNAbs, such as the CD4 binding site and the trimer apex, were preserved and remained accessible^14,20^. Recent studies clearly demonstrated that such nanoparticle vaccine candidates can potentiate the elicitation of autologous and even heterologous neutralizing antibody responses against Env^12,46,47^. These outcomes may root in the observation that the repetitive display of the antigen of interest on the surface of nanoparticles is favorable over non-repetitive antigens in the activation of antigen-specific B cells^19,48^. Furthermore, delivery via nanoparticles may protect vaccine antigens from rapid degradation by host enzymes after immunization^49,50^.

A viable approach is to present Env trimers at a high density on the surface of macromolecular platforms. Building on our previous research, we developed a two-step covalent attachment method to display Env trimers on nanoparticle surfaces in an orthogonal manner, closely mimicking the native viral morphology. Since aldehyde groups do not naturally occur in Env, a recombinantly introduced C-terminal aldehyde-tag on Env served as an ideal target for oxime attachment with aminooxy-containing reagents^51,52^. We used this method to functionalize Env with a C-terminal azide group via an aminooxy-linker. It was previously shown that the oxime ligation and subsequent conjugation to the surface of nanoparticles does not affect the antigenicity of Env trimers, since no significant reduction of broadly-neutralizing antibody binding (2G12, PG9, PGT145, b12) compared to untouched trimers was demonstrated^14^. To enable covalent binding via click chemistry, the L1-VLP surface must be functionalized with alkyne groups. Traditional click chemistry utilizes copper, Cu(I), to catalyze the 1,3-dipolar cycloaddition between alkyne and azide groups, resulting in the formation of a 1,2,3-triazole^53^. However, the presence of heavy metal ions such as copper may not be suitable for in vivo applications due to potential cytotoxicity and allergic reactions. To overcome this, we employed a biocompatible approach using the DBCO reagent, which allows the reaction with azides without the need of a copper catalyst. Additionally, L1-VLPs activated with the TFP-PEG4-DBCO Linker were traceable via UV-Vis spectroscopy at an absorption peak of 309 nm, enabling the evaluation of DBCO labeling efficiency.

Both in vitro (Fig. 4) and in vivo (Fig. 5) results showed enhanced activation of B cells and greater antibody production against the HIV Env protein when nanoparticles displaying Env on L1 scaffolds were used, significantly outperforming soluble antigen controls. Remarkably, even a single dose of these nanoparticles generated Env-specific IgG responses in mouse serum without external adjuvants, whereas soluble Env trimers alone failed to produce notable antibody levels (Fig. 5A). Upon subsequent booster injections, antibody responses elicited by the nanoparticle formulations considerably surpassed those induced by simultaneous administration of unconjugated Env trimers alongside free L1-VLPs (Fig. 5A). These findings emphasize the immunological benefits of covalently attaching antigens in a multivalent configuration rather than merely co-delivering soluble antigens with VLPs. The closely arranged, repetitive Env trimers on the L1-VLP surface facilitate effective cross-linking of BCRs, thereby enhancing B-cell activation and antigen internalization efficiency^14^. In line with current immunological concepts, our data confirmed that nanoparticle-formulated Env proteins elicited superior B-cell activation in vitro compared to soluble antigen forms, supporting a BCR cross-linking model. These multivalent engagements lower the activation threshold of B cells and enhance germinal center formation, as previously reported for similar nanoparticle-based antigen delivery systems^10,20,54^. Furthermore, intrinsic immune-stimulating properties of VLP platforms may inherently provide adjuvant effects, thus negating the necessity of external adjuvants and simplifying vaccine production and clinical implementation.

Chimeric nanoparticles formed by covalently linking L1 and Env proteins (L1-Env) induce immune responses against both HPV and HIV (Fig. 5), illustrating the potential of this vaccine strategy to simultaneously address the prevention of both viruses. Employing HPV L1-VLPs displaying HIV envelope epitopes confers several notable advantages. Primarily, a single vaccine may offer dual protection, particularly beneficial in regions with high HIV prevalence where enhanced HPV vaccination could significantly reduce HPV-related cancer risks, which are elevated in HIV-positive populations. Such a bivalent vaccine simplifies vaccination strategies (one vaccine, one cold-chain logistics, and one immunization schedule), potentially improving adherence and coverage for both diseases. We observed that our L1-Env nanoparticles significantly enhanced anti-Env antibody responses compared to the same amount of non-conjugated Env. Simultaneously, the L1 scaffold seemed to be partially shielded compared to non-conjugated L1-VLPs. Following the second (boost) immunization in the medium and high dose groups, we saw (i) a significant increase in anti-Env antibodies (Fig. 5A) and (ii) a reduction of L1 shielding, since L1-specific antibody levels were no longer significantly different from non-conjugated L1-VLPs (Fig. 5B). Therefore, it should be possible to balance the HIV Env and L1 antibody responses by adapting the Env density on the surface of the L1-VLPs further supporting the concept of a single vaccine providing dual protection.

The HIV Env glycoprotein is notoriously challenging as a vaccine antigen due to its substantial variability, intricate structure, dense glycosylation and evolutionary adaptations^37,55^. Consequently, traditional vaccine strategies frequently failed to induce potent antibody responses. Our research demonstrated that HPV-derived L1 nanoparticles, displaying structurally intact Env glycoproteins, significantly improved antibody responses in mice compared to traditional soluble Env trimers employed in earlier preclinical trials. This nanoparticle platform effectively amplified the immunogenic potential of surface-displayed antigens^56^. Notably, the focus of this study was to increase the HIV-Env antibody early after the immunization. Since wildtype mice cannot elicit neutralizing Tier-2 responses against HIV due to short CDRH3 domains of murine antibodies, we did not perform any neutralization assays. We and others demonstrated that vaccine delivery platforms displaying arrays of Env may be a critical component for the improvement of vaccine development efforts^16,57^. This and the excellent safety and immunogenicity record of HPV-VLPs, suggests that L1-Env nanoparticles are attractive vaccine candidates for further development.

## Conclusion

The current study demonstrates that HPV-16 L1-nanoparticles coated with HIV Env trimers elicit superior early humoral immune responses in mice compared to soluble, uncoupled Env. In vitro B cell activation experiments also show that the L1-Env nanoparticles can efficiently activate Env-specific BCRs, clearly outperforming uncoupled Env. Future research should focus on optimizing nanoparticle formulations to enhance specificity and efficacy, as well as conducting extensive in vivo studies to further validate our dual vaccine design. It is further possible in the future to utilize our platform for conjugation of germline-targeting Env constructs and other state-of-the-art vaccine candidates.

## Supplementary Information

Below is the link to the electronic supplementary material.

Supplementary Material 1

## Data Availability

The datasets used and/or analysed during the current study are available from the corresponding author on reasonable request.
