# Supplementary material for: Development and characterization of a human papillomavirus-based nanoparticle carrier for heterologous vaccine antigens
Source: Sci Rep. 2025 Sep 25;15:32927. doi: 10.1038/s41598-025-19009-3 (PMC12464271; doi:10.1038/s41598-025-19009-3)
Supplement: Supplementary file 1 — Supplementary Material 1 [file 41598_2025_19009_MOESM1_ESM.docx]

**Supplementary Figures (Wang et al.)**


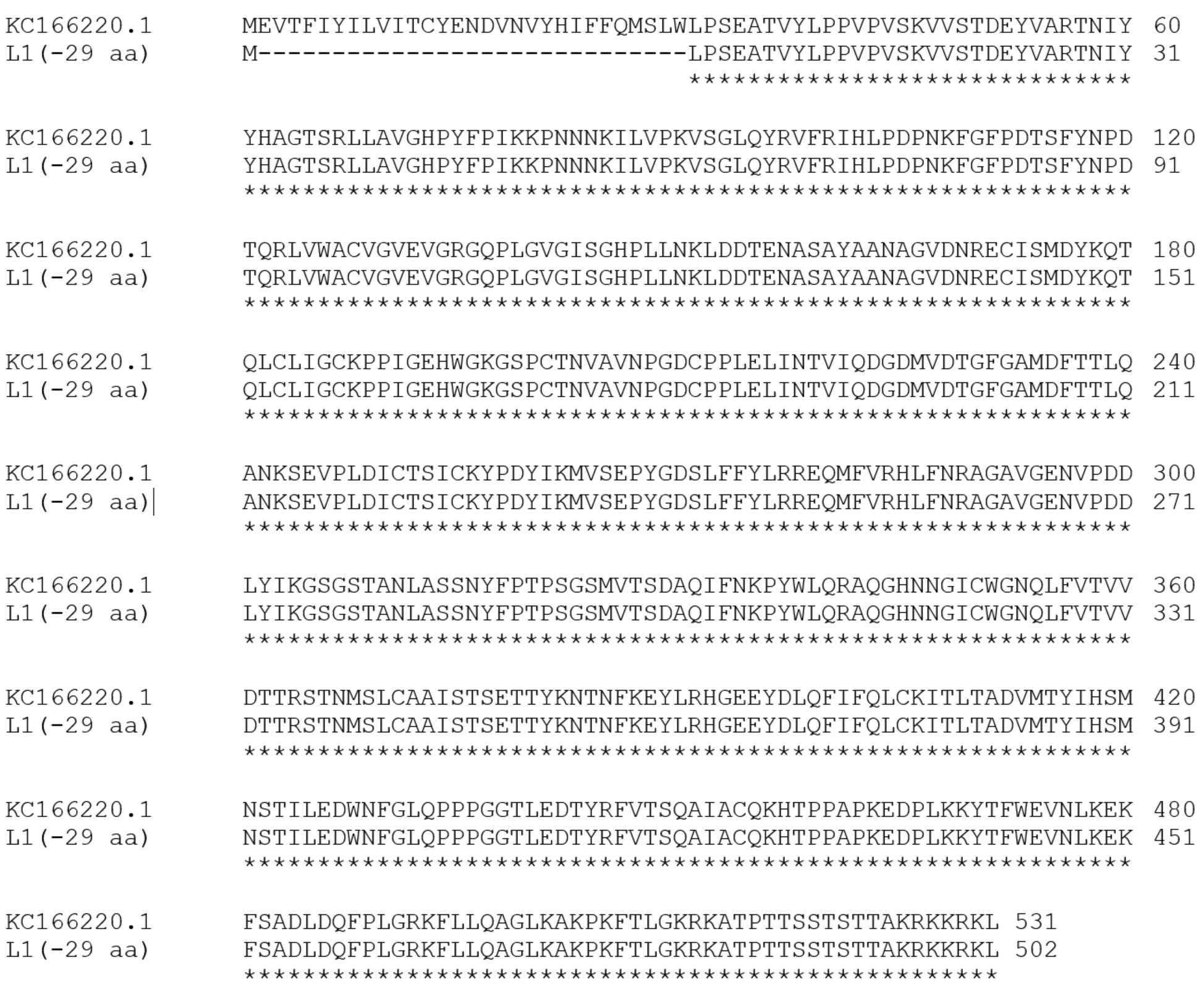


**Figure S1.** Amino acid sequence alignment of recombinant L1.

The amino acid sequence of the truncated L1 protein, that was used in this study, lacking twenty-nine N-terminal amino acids was aligned to the full-length reference sequence (GenBank accession number KC166220.1) via the Clustal Omega online tool ([www.ebi.ac.uk](http://www.ebi.ac.uk)).


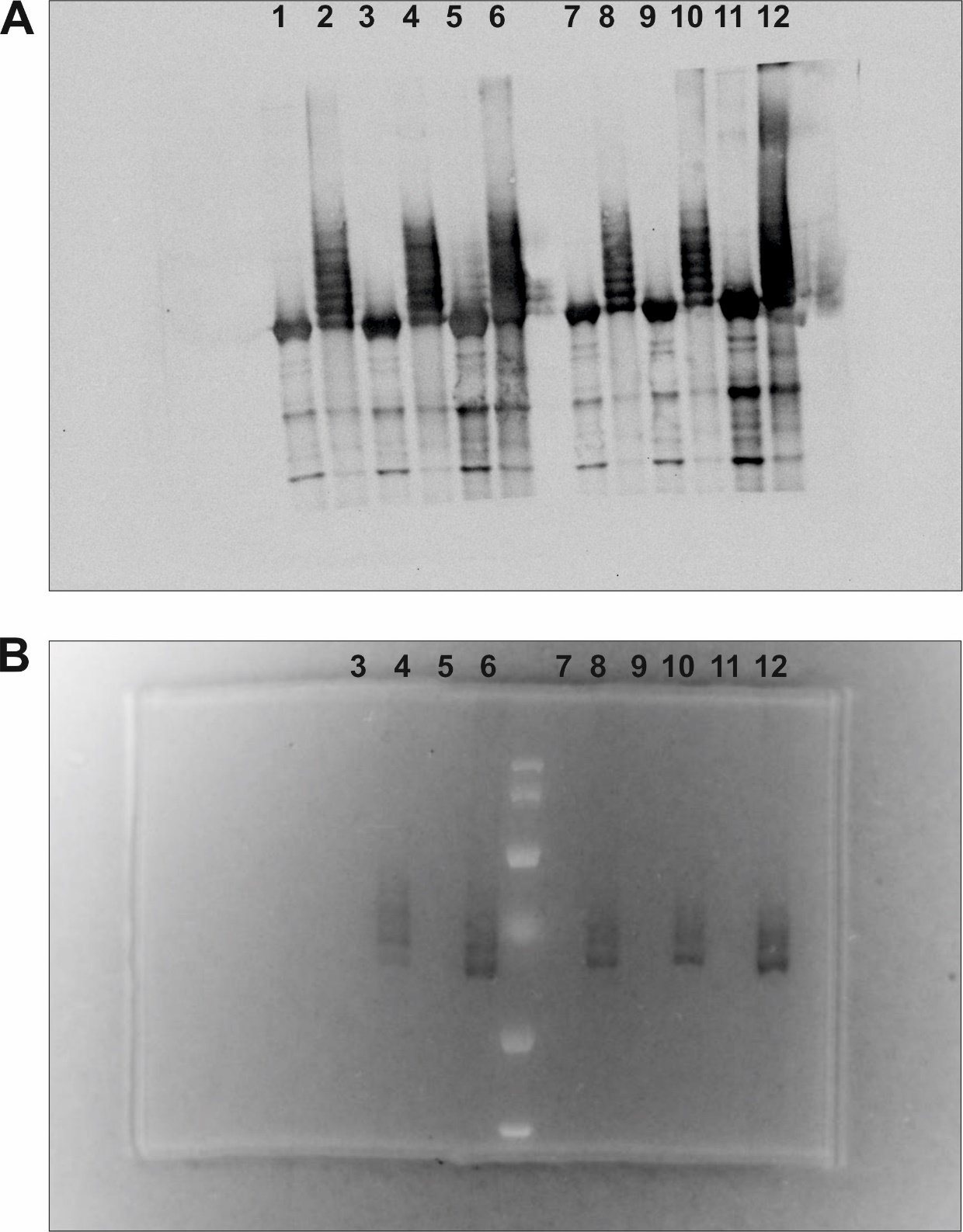


**Figure S2.** Analysis of peptide binding to L1-DBCO.

Western blot (A) and fluorescence gel (B) analysis of L1 proteins before and after DBCO activation. After Western Blot, the membrane was stained with anti-HPV-16 L1 antibody CAMVIR-1 and HRP-coupled secondary antibody. Lanes 1-6 correspond to one production batch, and lanes 7-12 to another. Within the same batch, samples were loaded onto SDS-PAGE (10 % bisacrylamide gels) at different amounts: 0.5 µg (lanes 1, 2, 7, 8), 1.25 µg (lanes 3, 4, 9, 10), and 2.5 µg (lanes 5, 6, 11, 12). Lanes 1, 3, 5, 7, 9, and 11: untreated L1 proteins; lanes 2, 4, 6, 8, 10, and 12: DBCO-activated L1 proteins. Following incubation with an N_3_- and FITC-labeled peptide, DBCO-activated L1 proteins exhibited detectable gel shifts compared to untreated controls. Lanes 9 and 10 were cropped and presented in Figure 3B.
